# A New Method for Node Fault Detection in Wireless Sensor Networks

**DOI:** 10.3390/s90201282

**Published:** 2009-02-24

**Authors:** Peng Jiang

**Affiliations:** Institute of Information and Control, Hangzhou Dianzi University, 310018, P.R. China; E-Mail: pjiang@hdu.edu.cn; Tel.: +86-571-86919131-512; Fax: +86-571-86919131

**Keywords:** Wireless sensor networks, node fault detection, improved DFD scheme

## Abstract

Wireless sensor networks (WSNs) are an important tool for monitoring distributed remote environments. As one of the key technologies involved in WSNs, node fault detection is indispensable in most WSN applications. It is well known that the distributed fault detection (DFD) scheme checks out the failed nodes by exchanging data and mutually testing among neighbor nodes in this network., but the fault detection accuracy of a DFD scheme would decrease rapidly when the number of neighbor nodes to be diagnosed is small and the node's failure ratio is high. In this paper, an improved DFD scheme is proposed by defining new detection criteria. Simulation results demonstrate that the improved DFD scheme performs well in the above situation and can increase the fault detection accuracy greatly.

## 1. Introduction

Wireless sensor networks (WSNs) are composed of massive, small and low-cost sensor nodes deployed in a monitoring region, forming a multi-hop self-organized network system through wireless communication. The target is to cooperatively sense, collect and process the information about objects in the coverage area, and then send it to the observer for processing and analyzing. It is a system with multi-functional and low energy consumption (see [1-4]).

Failed nodes may decrease the quality of service (Qos) of the entire WSN. It is important and necessary to study the fault detection methods for nodes in WSNs for the following reasons [5-6]:

1. Massive low-cost sensor nodes are often deployed in uncontrollable and hostile environments. Therefore, failure in sensor nodes can occur more easily than in other systems;
2. The applications of WSNs are being widened. WSNs are also deployed in some occasions such as monitoring of nuclear reactor where high security is required. Fault detection for sensor nodes in this specified application is of great importance;
3. It is troublesome and not practical to manually examine whether the nodes are functioning normally;
4. Correct information cannot be obtained by the control center because failed nodes would produce erroneous data. Moreover, it may result in collapse of the whole network in serious cases;
5. Nodes are usually battery-powered and the energy is limited, so it is common for faults to occur due to battery depletion.

WSN node faults are usually due to the following causes: the failure of modules (such as communication and sensing module) due to fabrication process problems, environmental factors, enemy attacks and so on; battery power depletion; being out of the communication range of the entire network.

The node status in WSNs can be divided into two types [7-8]: normal and faulty. Faulty in turn can be “permanent” or “static”. The so-called “permanent” means failed nodes will remain faulty until they are replaced, and the so-called “static” means new faults will not generated during fault detection. In [7,9], node faults of WSNs can be divided into two categories: hard and soft. The so-called “hard fault” is when a sensor node cannot communicate with other nodes because of the failure of a certain module (e.g., communication failure due to the failure of the communication module, energy depletion of node, being out of the communication range of entire mobile network because of the nodes' moving and so on). The so-called “soft fault” means the failed nodes can continue to work and communicate with other nodes (hardware and software of communication module are normal), but the data sensed or transmitted is not correct.

The remainder of the paper is organized as follows: In Section 2, related works in the area of fault detection in WSNs is reviewed. In Section 3, the DFD node fault detection scheme is introduced and the theory and realization of improved DFD node fault detection scheme is described in detail. The advantages and disadvantages of the two schemes are also analyzed. Simulation examples compare the fault detection accuracy of the two schemes with different network sizes, average number of neighbor nodes and failure ratios in Section 4. The paper is concluded in Section 5.

## 2. Related Work

In this section, we briefly review the related works in the area of fault detection in WSNs. The existence of faulty sensor measurements in WSNS will cause not only a degradation of the network quality of service, but also a huge burden on the limited energy. Article [10] investigates using the spatial correlation of sensor measurements to detect faults in WSNs. An approach of weighting the neighbors' measurement and presents a method to characterize the difference between sensor measurements are introduced. A weighted median fault detection scheme (WMFDS) is proposed and evaluated for both binary decisions and real number measurements.

In [11] the design of a distributed fault-tolerant decision fusion in the presence of sensor faults when the local sensors sequentially send their decisions to a fusion center is addressed. A collaborative sensor fault detection (CSFD) scheme is proposed to eliminate unreliable local decisions when performing distributed decision fusion. Based on the pre-designed fusion rule, assuming identical local decision rules and fault-free environments, an upper bound is established on the fusion error probability. According to this error boundary, a criterion is proposed to search the faulty nodes. Once the fusion center identifies the faulty nodes, all corresponding local decisions are removed from the computation of the likelihood ratios that are adopted to make the final decision.

Ref. [12] proposes a distributed solution for a canonical task in WSNs—the binary detection of interesting environmental events. They explicitly take into account the possibility of sensor measurement faults and develop a distributed Bayesian scheme for detecting and correcting such faults.

In [6], a taxonomy for classification of faults in sensor networks and the first on-line model-based testing technique are introduced. The technique considers the impact of readings of a particular sensor on the consistency of multi-sensor fusion. The sensor is most likely to be faulty if its elimination significantly improves the consistency of the results. A way to distinguish random noise is to run a maximum likelihood or Bayesian approach on the multi-sensor fusion measurements. If the accuracy of final results of multi-sensor fusion improve after running these procedure, some random noise should exist. To get a consistent mapping of the sensed phenomena, different sensors' measurements need to be combined in a model. This cross-validation-based technique can be applied to a broad set of fault models. It is generic and can be applied to an arbitrary system of sensors that use an arbitrary type of data fusion. However, this technique is centralized. Sensor node information must be collected and sent to the base station to conduct the on-line fault detection.

Article [13] proposes an agreement-based fault detection mechanism for detecting cluster-head failures in clustered Underwater Sensor Networks (UWSNs). Each cluster member is allowed to independently detect the fault status of its cluster head and at the same time a distributed agreement protocol is employed to reach an agreement on the fault status of the cluster head among multiple cluster members. The detection mechanism is based a TDMA MAC protocol used in the network and runs concurrently with normal network operation by periodically performing a distributed detection process at each cluster member. It makes use of the data periodically sent by a cluster head as the heartbeats for fault detection. A couple of forward and backward TDM frames are specially structured for enabling multiple cluster members to reach an agreement within two frames in each detection process. Moreover, a schedule generation scheme is also proposed for a cluster head to generate the transmission schedule of the forward and backward frames.

An energy efficient fault-tolerant detection scheme is proposed in [14] to introduce the sensor fault probability into the optimal event detection process. The optimal detection error was shown to decrease exponentially with the increase of the neighborhood size. They attempted to disambiguate events from both noise related measurement error and sensor fault and limit the effects of faulty sensor on the event detection accuracy. The measurement noise and sensor faults are likely to be stochastically unrelated, while event measurements are likely to be spatially correlated. The Bayesian detection scheme in [14] selects the minimum neighbors for a given detection error boundary such that the communication volume is minimized during the fault correction. Luo *et al*. in [14] did not explicitly attempt to detect faulty sensors, instead the schemes they proposed improve the event detection accuracy in the presence of faulty sensors.

Article [15] presents a distributed fault detection algorithm for wireless sensor networks. Each sensor node identifies its own status based on local comparisons of sensed data with some thresholds and dissemination of the test results. Time redundancy is used to tolerate transient faults in sensing and communication. To eliminate the delay involved in z time redundancy scheme a sliding window is employed with some storage for comparison of previous results.

In wireless sensor networks, multi-hop routing is commonly performed through a routing tree. Eventually, the routing tree needs to be rebuilt to accommodate failures, balance the energy consumption, or improve data aggregation. Most of the current solutions do not detect when the routing topology that needs to be rebuilt. Article [16] shows it is important to provide failure recovery and avoid unnecessary traffic when the routing topology needs to be rebuilt. It presents an inference engine, called Diffuse, designed to detect when the routing topology needs to be rebuilt based on different goals, such as to recover from routing failures, improve data aggregation, and balance the energy consumption. Diffuse approaches efficiently avoid unnecessary topology constructions. The authors use information/data fusion to detect routing failures, which is a different and promising approach. As stated in [17], information fusion techniques can reduce the amount of data traffic, filter noisy measurements, and make predictions and inferences about a monitored entity by exploiting the synergy among the available data.

The authors in [5] proposed and evaluated a localized fault detection scheme to identify the faulty sensors. Distributed fault detection (DFD) method has some shortcomings as follows: the fault detection accuracy will decrease rapidly in the case of the number of neighbor nodes to be diagnosed is all small and the node's failure ratio is high. High fault detection accuracy can be reached only when it is applied to the sensor network with many neighbors of nodes to be diagnosed. In this paper, an improved DFD scheme is proposed by defining new detection criterion to remedy the shortcomings above.

## 3. Theory and Realization of Improved DFD Fault Detection Scheme

### 3.1. Terms

Several terms used in this paper are explained as follows:

Fault detection accuracy: when determining the status of a node with a certain node fault detection scheme, the result can be divided into four cases which are: diagnosing the normal node (the node whose actual status is normal) as normal, the faulty node (the node whose actual status is faulty) as faulty, the normal node as faulty and the faulty node as normal. The sum of the probability of the two former cases is called fault detection accuracy.

Node's failure ratio: the probability of a node's failure in sensor network.

Neighbor node: The two nodes are neighbor nodes if the distance between them is within a single-hop's communication scope. The set of all neighbors of node *S_i_* is *Neighbor* (*S_i_*) and the total number of neighbors of node *S_i_* is noted as *Num*(*Neighbor* (*S_i_*)).

### 3.2. DFD Node Fault Detection Scheme

DFD node fault detection scheme proposed by Jinran Chen determines the status of node by testing among neighbor nodes mutually. For two neighbor nodes *S_i_* and *S_j_*, a test result *C_ij_* is produced by the data (such as temperature) sensed by each of them. The data at the moment *t* should be very close to each other because they are near, and the difference $d_{\text{ij}}^{t}$ between this data should not exceed a certain threshold *θ*_1_; besides, at another moment *t*+1, the difference of the data of the two neighbor nodes is $d_{\text{ij}}^{t+1}$, and the difference of $d_{\text{ij}}^{t+1}$ and $d_{\text{ij}}^{t}$ is $\Delta d_{\text{ij}}^{t}$ which should not exceed a certain threshold *θ*_2._ If one of these two conditions is not met, at least one of *S_i_* and *S_j_* is determined as a failure, and the test result *C_ij_* = 1, otherwise *C_ij_* = 0. For any node *S_i_*, its test result with each node in *Neighbor*(*S_i_*) can be obtained. If there are more than $\overset{\text{Num}(\text{Neighbor}(S_{i}))}{\;}/\underset{2}{\;}$ nodes whose test results are 1 in *Neighbor*(*S_i_*), then the initial detection status *T_i_* of node *S_i_* is possibly faulty (LT), otherwise, it may be possibly normal (LG). Thus, initial detection status of each node *S_i_* in the network is available.

For any node *S_j_* in *Neighbor*(*S_i_*), its actual status may be normal or faulty, so it may be not correct to determine the initial detection status of *S_i_* by the test result *C_ij_* which cannot be used to verify the status of *S_i_*. When the initial detection status of all nodes in the network is obtained, the following detection criterion is used for any node *S_i_*: for the nodes in *Neighbor*(*S_i_*) whose initial detection status is LG, subtract the number of nodes whose test result with *S_i_* is 0 from the number of nodes whose test result is 1. If the result is not less than $\overset{\text{Num}(\text{Neighbor}(S_{i}))}{\;}/\underset{2}{\;}$, then the status of *S_i_* is normal, otherwise, the status of *S_i_* is faulty.

### 3.3. Improved DFD Node Fault Detection Scheme

From the realization of DFD node fault detection scheme, for a normal node *S_normal_*, if the number of its neighbor nodes with initial detection status of LG is less than $\overset{\text{Num}(\text{Neighbor}(S_{\text{normal}}))}{\;}/\underset{2}{\;}$, then *S_normal_* is misdiagnosed as faulty, reducing the fault detection accuracy. The conditions of diagnosing the normal node as “normal” are too harsh in DFD node fault detection scheme. Besides, the node fault accuracy of DFD scheme will decrease rapidly when there are not many neighbors of the nodes to be diagnosed or the node's failure ratio of network is high.

The improved DFD node fault detection scheme proposed in this paper changes the detection criterion of DFD scheme as follows: for any node *S_i_* and the nodes in *Neighbor*(*S_i_*) whose initial detection status is LG, if the nodes whose test result with *S_i_* is 0 are not less than the nodes whose test result is 1, then the status of *S_i_* is normal (GD), otherwise, the status of *S_i_* is faulty (FT).

Improved DFD scheme takes the following steps:

- For node *S_i_* and any node *S_j_* in *Neighbor*(*S_i_*), set *C_ij_* as 0 and calculate $d_{\text{ij}}^{t}$.
  If $\left|d_{\text{ij}}^{t}\right|>\theta_{1}$, set *C_ij_* as 1 and turn to the next node in *Neighbor*(*S_i_*);
  If $\left|d_{\text{ij}}^{t}\right|\leq \theta_{1}$, calculate $\Delta d_{\text{ij}}^{t}$. If $\left|\Delta d_{\text{ij}}^{t}\right|>\theta_{2}$, set *C_ij_* as 1 and turn to the next node in *Neighbor*(*S_i_*);
  Repeat above steps until the test results of each node in *Neighbor*(*S_i_*) with *S_i_* are all obtained.
- If $\sum_{S_{j}\in \text{Neighbor}(S_{i})}C_{\text{ij}}<\overset{\text{Num}(\text{Neighbor}(S_{i}))}{\;}/\underset{2}{\;}$,set initial detection status *T_i_* of *S_i_* as possibly normal (LG), otherwise *T_i_* is possibly faulty (LT).
- *Num*(*Neighbor*(*S_i_*)*_T-LG_*) is the number of neighbor nodes of *S_i_* whose initial detection status is LG. If $(\sum_{S_{j}\in \text{Neighbor}(S_{i})\text{and}T_{j}=\text{LG}}C_{\text{ij}})<\overset{\text{Num}(\text{Neighbor}{\left(S_{i}\right)}_{T_{j}=\text{LG}})}{\;}/\underset{2}{\;}$, set the status of *S_i_* as normal (GD), otherwise it's faulty (FT).
- If there are no neighbor nodes of *S_i_* whose initial detection status is LG, and if the initial detection status *T_i_* of *S_i_* is LG, then set the status of *S_i_* as normal (GD), otherwise as faulty (FT).
- Check whether detection of the status of all nodes in network is completed or not. If it has been completed, then exit. Otherwise, repeat steps of (I), (II), (III) and (IV).

From the steps of improved DFD scheme, the status of node *S_i_* can also be correctly determined by improved DFD scheme when the number of nodes in *Neighbor*(*S_i_*) whose initial detection status is LG is small (node's failure ratio of network is high). Improved DFD scheme also can be applied in the sensor network where the neighbors of the nodes to be diagnosed are less.

We suppose the node's failure ratio is *p* and the average number of neighbors of each node is *k*. Set the probability of initially diagnosing the actual faulty (FT) node as possibly faulty (LT) is *P_flf_*, the actual normal (GD) node as possibly faulty (LT) is *P_glf_*, actual faulty (FT) node as possibly normal (LG) is *P_flg_*, and the actual normal (GD) node as possibly normal (LG) is *P_glg_*_,_ then:

(1) $$P_{\text{flf}}=p\sum_{i=0}^{m-1}C_{k}^{i}{\left(1-p\right)}^{k-i}p^{i}$$

(2) $$P_{\text{glf}}=(1-p)\sum_{j=0}^{m-1}C_{k}^{j}{\left(1-p\right)}^{j}p^{k-j}$$

(3) $$P_{\text{flg}}=p\sum_{j=0}^{m-1}C_{k}^{j}{\left(1-p\right)}^{j}p^{k-j}$$

(4) $$P_{\text{glg}}=(1-p)\sum_{i=0}^{m-1}C_{k}^{i}{\left(1-p\right)}^{k-i}p^{i}$$

Where, $m=\{\begin{array}{llll} \overset{k}{\;}/\underset{2}{\;}+1, & k & \text{is} & \text{even}\\ \overset{\left(k+1\right)}{\;}/\underset{2}{\;}, & k & \text{is} & \text{odd} \end{array}$. In formula (1) and (4), *i* is the number of failed nodes in the neighbors of the node to be diagnosed. According to the detection criterion of DFD scheme, the faulty nodes (normal nodes) can be initially diagnosed as possibly faulty (possibly normal) when *i* is not larger than half of the number of neighbors of the node to be diagnosed which is *m*-1. Simultaneously, in formula (2) and (3), *j* is the number of normal nodes in the neighbors of the node to be diagnosed. According to the detection criterion of DFD scheme, the normal nodes (faulty nodes) will be initially diagnosed as possibly faulty (possibly normal) when *j* is not larger than half of the number of neighbors of the node to be diagnosed which is *m*-1.

In improved DFD scheme, the possibility of diagnosing the actual faulty node as normal is:

(5) $$P_{\text{FG}}=p\sum_{x=1}^{k}C_{k}^{x}(\sum_{y=0}^{n-1}C_{x}^{y}P_{\text{glg}}^{y}P_{\text{flg}}^{x-y})(\sum_{a=0}^{k-x}C_{k-x}^{a}P_{\text{glf}}^{a}P_{\text{flf}}^{k-x-a})+P_{\text{flg}}(\sum_{a=0}^{k}C_{k}^{a}P_{\text{glf}}^{a}P_{\text{flf}}^{k-a})$$

The possibility of diagnosing the actual normal node as faulty is:

(6) $$P_{\text{GF}}=(1-p)\sum_{x=1}^{k}C_{k}^{x}(\sum_{y=0}^{n-1}C_{x}^{y}P_{\text{glg}}^{y}P_{\text{flg}}^{x-y})(\sum_{a=0}^{k-x}C_{k-x}^{a}P_{\text{glf}}^{a}P_{\text{flf}}^{k-x-a})\;+P_{\text{glf}}(\sum_{a=0}^{k}C_{k}^{a}P_{\text{glf}}^{a}P_{\text{flf}}^{k-a})$$

The possibility of diagnosing the actual normal node as normal is:

(7) $$P_{\text{GG}}=(1-p)\sum_{x=1}^{k}C_{k}^{x}(\sum_{z=0}^{n-1}C_{x}^{z}P_{\text{glg}}^{x-z}P_{\text{flg}}^{z})(\sum_{a=0}^{k-x}C_{k-x}^{a}P_{\text{glf}}^{a}P_{\text{flf}}^{k-x-a})+P_{\text{glg}}(\sum_{a=0}^{k}C_{k}^{a}P_{\text{glf}}^{a}P_{\text{flf}}^{k-a})$$

The possibility of diagnosing the actual faulty node as faulty is:

(8) $$P_{\text{FF}}=p\sum_{x=1}^{k}C_{k}^{x}(\sum_{z=0}^{n-1}C_{x}^{z}P_{\text{glg}}^{x-z}P_{\text{flg}}^{z})(\sum_{a=0}^{k-x}C_{k-x}^{a}P_{\text{glf}}^{a}P_{\text{flf}}^{k-x-a})+P_{\text{flf}}(\sum_{a=0}^{k}C_{k}^{a}P_{\text{glf}}^{a}P_{\text{flf}}^{k-a})$$

where, $n=\{\begin{array}{llll} \overset{x}{\;}/\underset{2}{\;}+1, & x & \text{is} & \text{even}\\ \overset{\left(x+1\right)}{\;}/\underset{2}{\;}, & x & \text{is} & \text{odd} \end{array}$. In formulas (5) to (8), *x* is the number of nodes in the neighbors of the node to be diagnosed which is initially diagnosed as possibly normal (LG). In formulas (5) and (6), *y* is the number of actual normal (GD) nodes initially diagnosed as possibly normal (LG) in *x* nodes. According to the detection criterion of the improved DFD scheme, mistakes will be made to the detection of status of nodes when *y* is not larger than half of *x*, *n*-1. Simultaneously, in formulas (7) and (8), *z* is the number of actual faulty (FT) nodes initially diagnosed as possibly normal (LG) in *x* nodes. According to the detection criterion of the improved DFD scheme, the actual status can be diagnosed only when *z* is not larger than half of *x*, *n*-1. In formulas (5) to (8), the item at the right of plus sign is the probability of diagnosing the status of nodes by improved DFD scheme when there is no neighbor of the node to be diagnosed which is initially diagnosed as possibly normal (LG).

From formulas (7) and (8), the fault detection accuracy of improved DFD scheme is:

(9) $$P_{\text{improved}\;\text{DFD}}=P_{\text{GG}}+P_{\text{FF}}$$

The fault detection accuracy of DFD scheme is:

(10) $$\begin{array}{l} \begin{array}{ll} P_{\text{DFD}} & =1-p\sum_{x=m}^{k}C_{k}^{x}(\sum_{l=0}^{t-1}C_{x}^{l}P_{\text{glg}}^{l}P_{\text{flg}}^{x-l})(\sum_{b=0}^{k-x}C_{k-x}^{b}P_{\text{glf}}^{b}P_{\text{flf}}^{k-x-b})\\ & +(1-p)\sum_{x=m}^{k}C_{k}^{x}(\sum_{l=0}^{t-1}C_{x}^{l}P_{\text{glg}}^{x-l}P_{\text{flg}}^{l})(\sum_{b=0}^{k-x}C_{k-x}^{b}P_{\text{glf}}^{b}P_{\text{flf}}^{k-x-b}) \end{array}\\ \; \end{array}$$

where, $t=\{\begin{array}{llll} \overset{\left(x-m\right)}{\;}/\underset{2}{\;}+1, & x-m & \text{is} & \text{even}\\ \overset{\left(x-m+1\right)}{\;}/\underset{2}{\;}, & x-m & \text{is} & \text{odd} \end{array}$. *x* is the number of nodes in the neighbors of the node to be diagnosed which is initially diagnosed as possibly normal (LG). DFD can diagnose the actual normal (GD) node as normal only when *x* is larger than half of the number of neighbors of the node to be diagnosed which is *m*-1. $(1-p)\sum_{x=m}^{k}C_{k}^{x}(\sum_{l=0}^{t-1}C_{x}^{l}P_{\text{glg}}^{x-l}P_{\text{flg}}^{l})(\sum_{b=0}^{k-x}C_{k-x}^{b}P_{\text{glf}}^{b}P_{\text{flf}}^{k-x-b})$ is the possibility of diagnosing the actual normal (GD) node as normal (GD) by DFD scheme. $1-p\sum_{x=m}^{k}C_{k}^{x}(\sum_{l=0}^{t-1}C_{x}^{l}P_{\text{glg}}^{l}P_{\text{flg}}^{x-l})(\sum_{b=0}^{k-x}C_{k-x}^{b}P_{\text{glf}}^{b}P_{\text{flf}}^{k-x-b})$ means the possibility of diagnosing the actual faulty (FT) node as faulty by DFD scheme.

For different number of neighbor nodes *k* and nodes' failure ratio *p*, the fault detection accuracy of DFD and improved DFD scheme calculated by formulas (1) ∼ (10) are shown in Tables 1 and 2, respectively, from which we can see that the fault detection accuracy of the two schemes decrease with the decreasing of *k* and increasing of *p*. For the same *k* and *p*, the fault detection accuracy of improved DFD scheme is obviously higher than DFD scheme. Besides, improved DFD scheme can also keep high fault detection accuracy even with high node's failure ratio and small average number of neighbor nodes.

## 4. Simulation Examples

**Figure 1.**
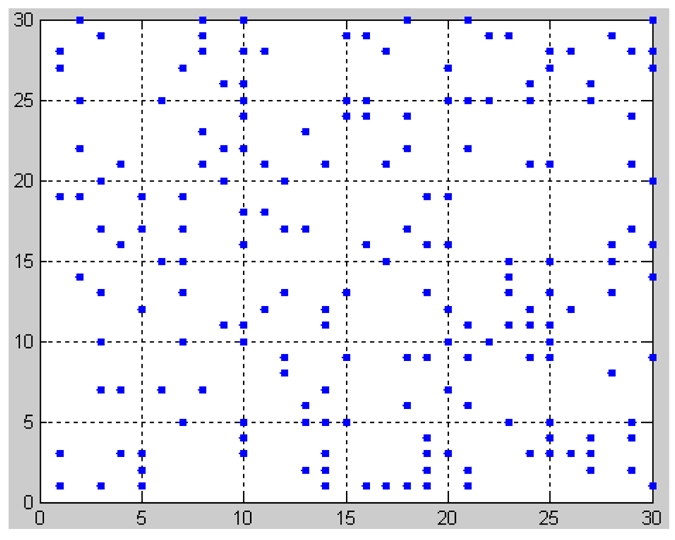
The sensor network with 200 randomly deployed nodes.

The improved DFD scheme will be applied in a real wireless sensor network system. It is expensive to run schemes on the hardware of the system, so the feasibility and accuracy of the schemes should be verified before being applied. Therefore, simulation becomes the best alternative way of testing, evaluating and verifying. We programmed the DFD scheme and improved DFD scheme using Visual C++ and Matlab. We compared the change of fault detection accuracy of the two schemes with varying node failure ratios for different average numbers of neighbor nodes. Two hundred nodes are randomly deployed in the network, as shown in Figure 1.

**Figure 2.**
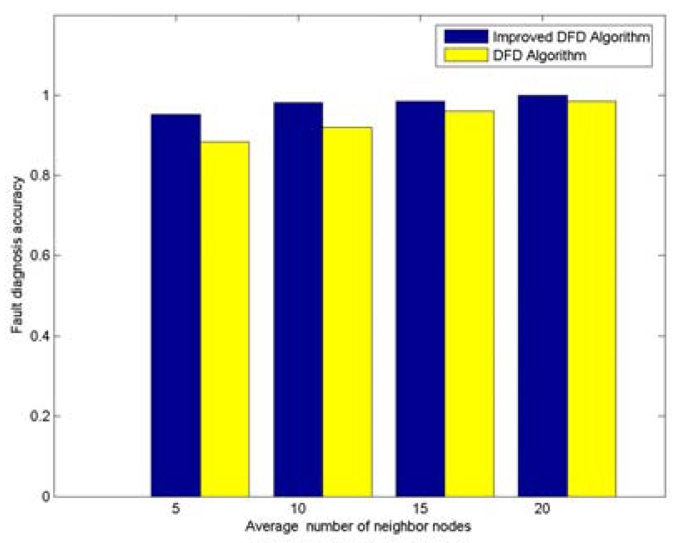
The trend of node fault detection accuracy with various average numbers of neighbor nodes when 200 nodes are deployed and the node's failure ratio is 0.3.

**Figure 3.**
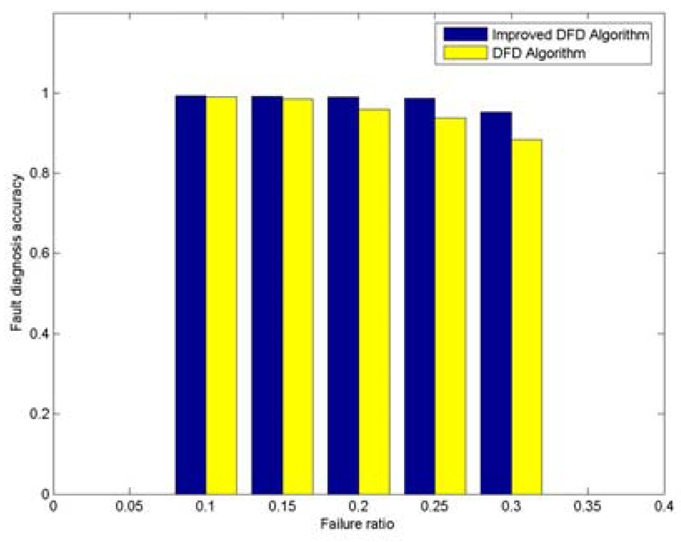
The trend of node fault detection accuracy with various node failure ratios when 200 nodes are randomly deployed and the average numbers of neighbor nodes is 5.

With 200 randomly deployed nodes, the node fault detection accuracy trend with various average numbers of neighbor nodes is shown in Figure 2. The node failure ratio is taken to be 0.3. It can be seen that the fault detection accuracy of DFD and improved DFD scheme increase with the increasing of average number of neighbor nodes, and the improved DFD scheme outperforms the DFD scheme. Similarly, the trend of node fault detection accuracy with various node failure ratios is also analyzed when 200 nodes are randomly deployed and the average numbers of neighbor nodes is 5. Figure 3 indicates that the fault detection accuracy of the DFD and the improved DFD scheme decreases with the increase in the node failure ratio and the improved DFD scheme also outperforms the DFD scheme.

**Figure 4.**
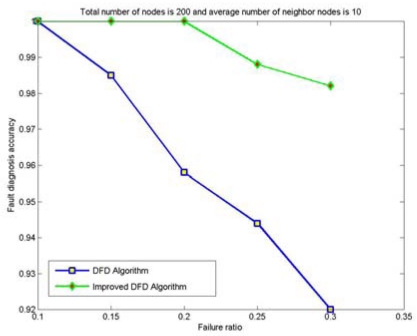
The node fault detection accuracy with various node failure ratios when 200 nodes are randomly deployed and the average numbers of neighbor nodes is 10.

**Figure 5.**
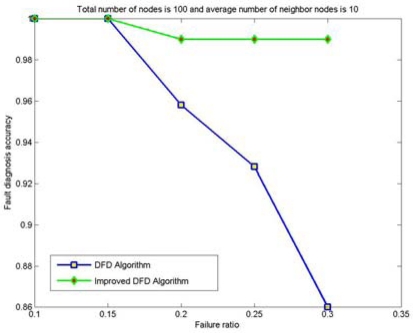
The node fault detection accuracy with various node failure ratios when 100 nodes are randomly deployed and the average numbers of neighbor nodes is 10.

**Figure 6.**
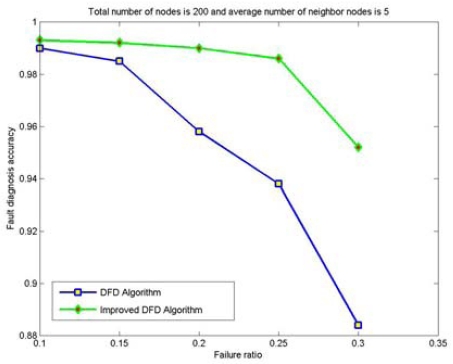
The node fault detection accuracy with various node failure ratios when 200 nodes are randomly deployed and the average numbers of neighbor nodes is 5.

**Figure 7.**
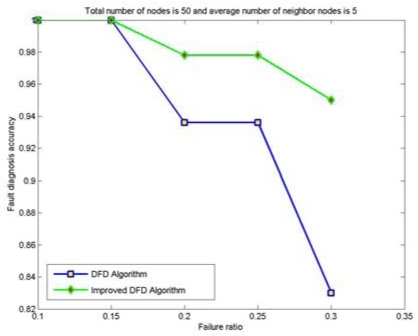
The node fault detection accuracy with various node failure ratios when 50 nodes are randomly deployed and the average numbers of neighbor nodes is 5.

The node fault detection accuracy of the DFD and the improved DFD scheme for different network size is also analyzed and compared. Figures 4 to 7 show the trend of node fault detection accuracy with various node failure ratios with different total number of nodes deployed and average number of neighbor nodes. Figure 4 shows the situation when 200 nodes are deployed and the average number of neighbor nodes is 10. Figure 5, 6 and 7 display the situations with 100 nodes deployed and 10 average neighbor nodes, 200 nodes deployed and 5 average neighbor nodes, and 50 nodes deployed and 5 average neighbor nodes, respectively.

From Figured 4 to 7, we can see that for the same total number of nodes deployed, the average number of neighbor nodes and the nodes' failure ratio, the improved DFD scheme distinctly outperforms the DFD scheme. The fault detection accuracy of the DFD scheme sharply decreases with an increase of the nodes' failure ratio. However, the improved DFD scheme retains a high fault detection accuracy.

Comparing Figure 4 to Figure 5 and Figure 6 to Figure 7, we can see that node fault detection accuracy of both schemes decreases with the reduction of network size with same average number of neighbor nodes and node failure ratios. The improved DFD scheme performs better than the DFD scheme for node fault detection.

In Figure 7, the node fault detection accuracy of the DFD scheme was reduced to 83%, while the improved DFD scheme can remain above 94%, when the total number of nodes is 50, average number of neighbor nodes is 5 and the node failure ratio is 30%. It indicates that the improved DFD scheme can be better applied to smaller scale wireless sensor networks with less neighbor nodes.

Therefore, compared with the DFD scheme, the improved DFD scheme greatly increases the node fault detection accuracy and high fault detection accuracy can be obtained even with high node failure ratios and small average number of neighbor nodes.

## 5. Conclusions

For the node whose actual status is normal, if the number of nodes which is initially diagnosed as possibly normal (LG) in its neighbor nodes is less than half of the total neighbor nodes, the DFD node fault detection scheme will misdiagnose the normal node as faulty. Modification is made to the detection criterion of DFD scheme and an improved DFD scheme is proposed to address this shortcoming. Simulation results show that the fault detection accuracy of the improved DFD scheme outperforms the DFD scheme for different average numbers of neighbor nodes and node failure ratios. The improved DFD scheme can also be applied to wireless sensor networks where there are less neighbor nodes and the node failure ratio is higher.

## Figures and Tables

**Table 1.** Fault detection accuracy of DFD scheme.

**Node's failure ratio***ρ*	**Average number of neighbor nodes *k***				
	**5**	**10**	**15**	**20**
0.1	0.998	1	1	1
0.15	0.985	0.992	1	1
0.2	0.962	0.976	0.993	1
0.25	0.935	0.955	0.981	0.992
0.3	0.873	0.917	0.968	0.976

**Table 2.** Fault detection accuracy of improved DFD scheme.

**Node's failure ratio***p*	**Average number of neighbor nodes *k***			
	**5**	**10**	**15**	**20**
0.1	1	1	1	1
0.15	0.997	1	1	1
0.2	0.992	0.997	1	1
0.25	0.985	0.992	1	1
0.3	0.964	0.986	0.999	1
